# The readability and quality paradox: Comparing ChatGPT, Gemini, and Perplexity outputs on pediatric chest pain queries

**DOI:** 10.1371/journal.pone.0359170

**Published:** 2026-09-25

**Authors:** Hikmet Kıztanır, Ebru Çetin Özbek

**Affiliations:** 1 Division of Pediatric Cardiology, Department of Pediatrics, Recep Tayyip Erdogan University Faculty of Medicine, Rize, Turkey; 2 Department of Pediatrics, Derince Training and Research Hospital, University of Health Sciences, Kocaeli, Turkey; The First Affiliated Hospital of Sun Yat-sen University, CHINA

## Abstract

Pediatric chest pain represents a frequently encountered symptom in emergency departments and outpatient clinics throughout childhood, often generating substantial parental anxiety. This study aims to comparatively analyze the readability, information quality, and scientific reliability of textual contents generated by the artificial intelligence (AI) chatbots ChatGPT, Gemini, and Perplexity regarding pediatric chest pain, utilizing multidimensional analytical indices. Out of the top 25 queries with the highest global search volume on Google Trends, 17 unique keywords meeting the predefined inclusion criteria were filtered on June 1, 2026. These inquiries were directed to all three AI platforms in distinct, independent user sessions, yielding a total of 51 textual responses. Linguistic readability levels were calculated across six separate digital interfaces using the FKGL, FRES and other formulas, and the results were benchmarked against the sixth-grade reading level” threshold. Scientific reliability was audited via the Modified DISCERN and JAMA benchmarks, while content quality was examined using the GQS and EQIP instruments. The median readability scores computed across all three AI models were found to be statistically and significantly above the targeted sixth-grade comprehension threshold, indicating a high level of difficulty (p < 0.001). Post-hoc pairwise evaluations revealed that ChatGPT and Gemini offered linguistically more accessible and structurally less complex textual architectures; conversely, Perplexity exhibited a significantly more difficult and academic linguistic framework (p < 0.0167). On the other hand, regarding content quality and source credibility, Perplexity demonstrated remarkably superior and more optimized scores across all evaluated instruments, namely GQS (p = 0.002, p = 0.001), JAMA (p < 0.001, p < 0.001), mDISCERN (p = 0.005, p = 0.001), and EQIP (p = 0.003, p < 0.001), compared directly to both ChatGPT and Gemini, respectively. Although generative AI formulations harbor considerable potential to provide extensive data on pediatric chest pain, the sophisticated, university-level linguistic architecture of these texts poses a substantial access barrier for individuals who lack proficient digital health literacy skills. While Perplexity achieved the performance closest to the “gold standard” in terms of informational accuracy, no model is currently mature enough to substitute for a professional medical consultation due to observed citation biases and omissions. It is of paramount importance that clinicians actively guide families to scrutinize online health data through a critical lens.

## Introduction

Pediatric chest pain stands as one of the most widespread clinical presentations within childhood emergency care and outpatient clinics. Although the vast majority of presentations are linked to benign etiologies, it remains a clinical scenario that warrants meticulous exploration due to the rare possibility of it being the initial manifestation of critical cardiac pathologies. While the underlying etiology in these young patients predominantly stems from musculoskeletal, psychogenic, or idiopathic conditions, cardiac-related factors constitute an exceedingly low percentage. Nevertheless, the profound apprehension it provokes in both the patients and their parents imposes a distinct and heavy administrative and clinical workload on healthcare delivery systems. Comprehensive history taking and physical examinations typically serve as the primary pillars for differential diagnosis during clinical assessments; however, the overutilization of redundant, advanced diagnostic testings persists as a prominent clinical challenge [1–3]. Viewed from this perspective, pediatric chest pain represents a major subject of interest in terms of both daily clinical practice and broader healthcare resource utilization.

In recent years, the deployment of artificial intelligence (AI) technologies across the medical domain has experienced an exponential rise. Parallel to this paradigm shift, AI-driven platforms have become increasingly prevalent in creating digital health content and facilitating patient education regarding pediatric symptoms. Conversational chatbots, in particular, stand out as primary resources through which parents query their children's symptoms, harvest information regarding potential diagnoses, and potentially alter their decisions regarding when to seek formal medical care [4]. In widespread clinical scenarios that possess an expansive etiological spectrum—such as pediatric chest pain—the accuracy and scientific trustworthiness of the information generated by these AI models assume critical importance. Nonetheless, a definitive scientific consensus regarding the technical adequacy and clinical suitability of such AI-generated literature is currently lacking in existing research [5–7]. Misleading or incomplete digital counseling can trigger unwarranted panic, prompt inappropriate medical admissions, and induce dangerous delays in necessary clinical interventions.

The clinical value of health-related information relies not only on its factual precision but also on its comprehensibility to the intended audience. Readability is scrutinized through various validated analytical metrics that assess how well a text matches the reader’s age, educational background, and linguistic skills. For this purpose, readability algorithms like Flesch-Kincaid, Gunning Fog, and SMOG are widely utilized to categorize written materials according to corresponding educational milestones. The American Medical Association (AMA), the National Institutes of Health (NIH), and the U.S. Department of Health and Human Services collectively advocate that patient-facing educational resources should be composed at or below a sixth-grade literacy level [8–10]. Considering that parents regularly investigate pediatric medical issues via web-based channels, delivering this information in an explicit, straightforward, and easily digestible language is crucial for optimizing the clinical decision-making pathway [11,12].

To date, investigations evaluating the health literature produced by AI-backed conversational agents specifically for pediatric chest pain remain highly constrained. The existing body of literature emphasizes an urgent need for more comprehensive, structured, and systematic evaluations focusing on both content quality and linguistic readability. Consequently, this investigation seeks to systematically audit the texts generated by various AI-powered chatbots regarding pediatric chest pain from the dual viewpoints of quality and readability. The anticipated insights are intended to establish a clearer analytical framework regarding the dependability of digital health data, ultimately contributing to the advancement of patient- and parent-centered communication models in pediatric medicine.

## Materials and methods

### Ethical statement

The design of this investigation did not involve any interventional or observational procedures on human participants or live subjects. Consequently, in accordance with established institutional and scientific guidelines, this study falls outside the category of research requiring formal ethics committee approval.

### Research design and data collection

To eradicate individual user biases and potential algorithmic distortions during data extraction, all browser cookies and search histories were completely cleared prior to pulling data. To prevent geographical location manipulation and minimize historical data bias, no Virtual Private Network (VPN) was used, and all queries were processed through the Incognito interface of the Google platform. Data for the keyword “Pediatric chest pain” were extracted from the trends mode of Google database on June 1, 2026, to analyze worldwide search trends and geographical distributions. Worldwide statistics from 2004 up to the exact moment of data retrieval were monitored utilizing the “most relevant” filter, and the top 25 most popular search terms along with their regional density metrics were recorded [13]. To eliminate prompt-engineering bias and evaluate the default baseline outputs of the AI platforms, the filtered search terms extracted from Google Trends were entered directly into the LLMs as verbatim queries (e.g., ‘chest pain in children,’ ‘pediatric chest pain’) rather than expanded conversational prompts (e.g., ‘My child has chest pain, what should I do?’). This standardized input approach ensured that the generated responses reflected the models’ raw, unguided linguistic complexity and content quality when handling direct medical search queries. Google was chosen as the primary data repository for this study because it held a dominant 82.24% global market share as of October 2025, thus offering the most comprehensive data pool available [14].

### AI analysis protocol

This study qualitatively examined the outputs generated by three globally prominent large language models (LLMs)—ChatGPT, Perplexity, and Gemini—in response to “Pediatric chest pain” keywords. The designated search terms were fed into the platforms as structured English queries [6,13]. To ensure the models were not influenced by prior query histories and to circumvent systematic bias, completely independent, fresh, and reset user sessions were opened for each specific keyword combination. The gathered text responses were classified and logged into a database based on medical accuracy, clinical reliability, and standard readability metrics. Adhering to open science methodology, the analyzed raw data and the full unaltered responses from the AI models are publicly accessible via the corresponding digital repository link: https://zenodo.org/records/22212297.

The evaluation utilized the current free edition of ChatGPT (GPT-5.5). Free versions of Gemini 3.5 Flash, and the Perplexity’s web-grounded Sonar model also utilized [9,15]. Redundant phrases, synonymous terms, and expressions falling outside the clinical scope of “Pediatric chest pain” served as exclusion criteria. Conversely, English keywords directly tied to pediatric chest pain that demonstrated literature relevance constituted the inclusion criteria [13,16].

### Textual readability and linguistic complexity assessment

To cross-verify the semantic and structural readability of the AI-generated responses, optimize data reliability, and eliminate software anomalies, a cross-check validation was executed across two separate digital testing platforms. To address potential algorithmic variations, syllable-tokenization discrepancies, and software-specific anomalies inherent in automated text parsers, a dual-platform cross-validation protocol was executed. All raw textual outputs were processed independently through two widely utilized digital engines: readabilityformulas.com (Calculator 1) and online-utility.org (Calculator 2). Using a single web tool or codebase risks introducing platform-specific measurement bias. Therefore, integrating data across two independent calculation interfaces and utilizing their arithmetic mean ensured cross-platform verification, minimized technical variance, and enhanced the overall methodological robustness and reproducibility of the readability indices.Textual density and structural difficulty were quantified using six foundational readability indices validated in academic literature: Coleman-Liau Index (CLI), Flesch Reading Ease Score (FRES), FKGL, GFOG, Automated Readability Index (ARI), and SMOG [17]. Final profiles were benchmarked against the “sixth-grade reading level” standard endorsed by the AMA and NIH for public health literature. Within this analytical framework, an 80.0 score was set as the minimum success threshold for the FRES parameter, while a grade level of 6.0 served as the target success criterion for the FKGL, GFOG, CLI, ARI, and SMOG indices [5,6].

### Scientific reliability and content quality auditing

To evaluate the academic rigor and consistency of the AI-generated medical documents using objective parameters, two foundational evaluation instruments widely recognized in the literature were validated. Initially, the Modified DISCERN Scale was used to assess data source reliability across five structured dimensions. This protocol scored attributes such as referencing, currency, methodological transparency, handling of controversial medical themes, and objectivity on a scale from 0 to 5. Higher cumulative scores directly indicated greater reliability and academic quality [15]. The methodological validity of standardized evaluation tools like DISCERN and JAMA is well-documented in earlier medical informatics literature [13]. The second reliability benchmark utilized the JAMA ranking guidelines, evaluating each text output against four core publishing ethics tenets: currency, authorship, disclosure, and attribution [5,6]. The final absolute score of the JAMA (maximum of 4 points) illustrated the content's alignment with academic requirements. High scores achieved through this analysis indicated the platform's methodological compliance with ethical publishing practices and evidence-based medicine norms [6,13].

### Analysis of content quality

To assess the overall standards and clinical utility of the retrieved medical texts for consumers, the widely validated Global Quality Score (GQS] was utilized [18]. This tool categorizes text outputs using a 5-point Likert scale, where 1 point represents poor quality, insufficient data lacking any medical value for patients, and 5 points denotes highly reliable, comprehensive materials that offer direct clinical utility [19,20]. The analytical strength and validity of the GQS tool across diverse digital health studies have been verified by previous scientific research [21,22]. Another quality control mechanism integrated into the study was the EQIP (Ensuring Quality Information for Patients) scale. This tool appraises the extracted answers through a structured set of 20 criteria, marking each item as “yes” (1 point), “partially” (0.5 points), or “no” (0 points). The final quality index was calculated as a percentage by dividing the total score by the number of applicable criteria and multiplying by 100. The resulting percentage data classified content quality into four hierarchical categories: 76–100% designated high-quality, “well-written” texts; 51–75% indicated structurally sound content containing minor issues; 26–50% comprised materials with significant flaws; and 0–25% represented severely problematic contents regarding clinical safety.

## Results

**Fig 1:**
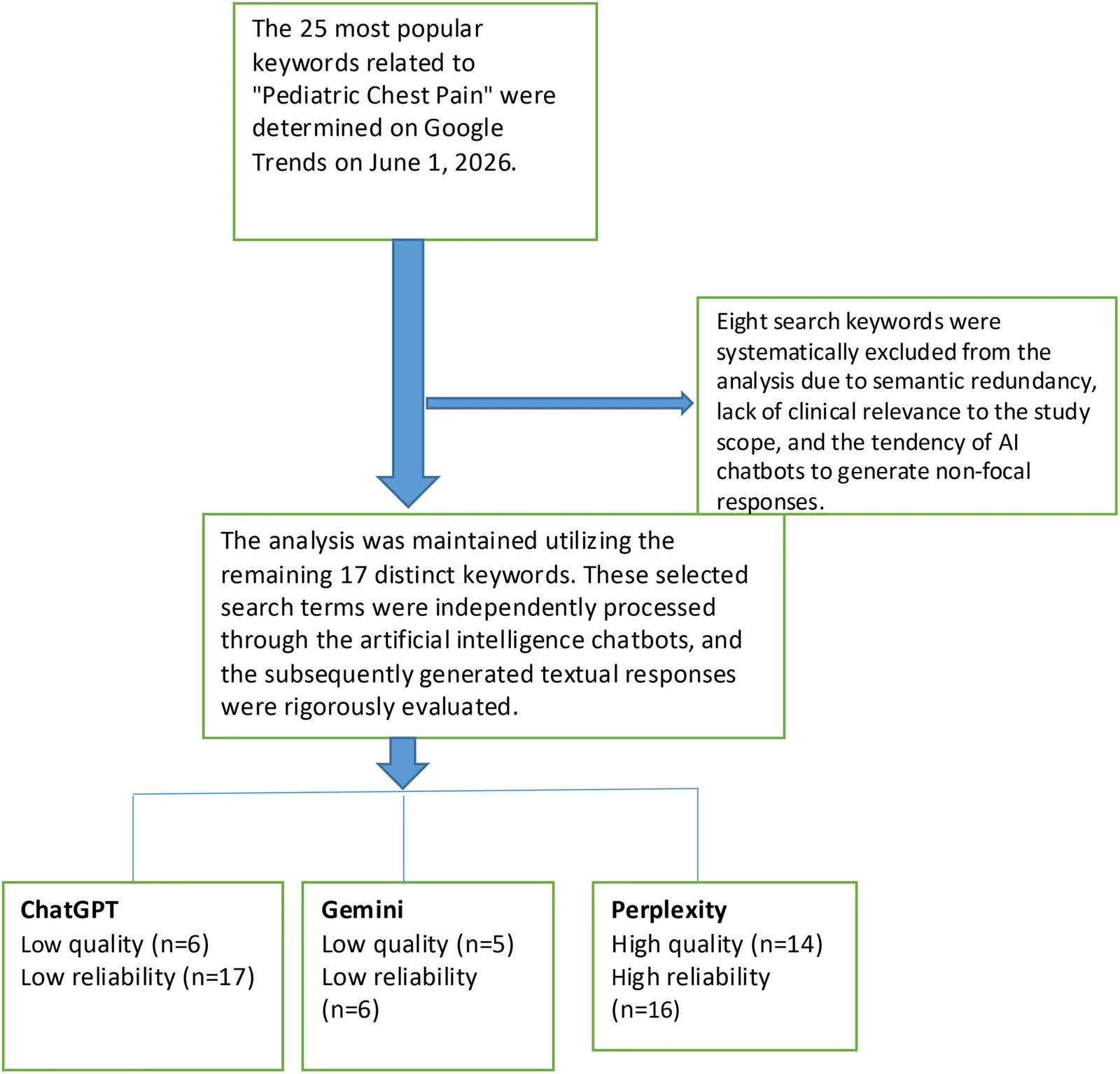
Flow Diagram of the Keywords.

The Google Trends database was analyzed to map digital information-seeking patterns and user behavior regarding the keyword “pediatric chest pain.” From an initial pool of 25 potential search terms, 8 were excluded based on predefined criteria, such as semantic redundancy, lack of alignment with the clinical boundaries of the study, or potential to generate off-context outputs in AI models [5,6,13]. Exactly 8 terms were excluded based on predefined methodological criteria: Semantic Redundancy (n = 4): Lexical variations or morphological duplicates that yielded identical clinical topics were consolidated to prevent data duplication (e.g., ‘chest pain child’ and ‘child chest pain’ were excluded in favor of the primary high-volume term ‘chest pain in children’). Out-of-Scope/Clinical Irrelevance (n = 3): Queries targeting non-pediatric demographics or distinct non-cardiorespiratory pediatric symptoms were removed (e.g., ‘adult chest pain causes,’ ‘pediatric cough’). Off-Context Generative Risk (n = 1): Broad anatomical terms that triggered generic educational descriptions rather than clinical/symptom-oriented evaluations were excluded (e.g., ‘chest anatomy’). The remaining 17 distinct terms met all inclusion criteria by directly focusing on pediatric chest pain presentation, differential diagnoses, or related cardiac/non-cardiac etiology. Following this filtering process, the remaining 17 unique keywords most relevant to the study scope were included in the analytical workflow (Fig 1).

These 17 specific query combinations were independently submitted to each of the three AI platforms, generating a total dataset of 51 text files for evaluation. The resulting documents were independently evaluated for readability, content quality, and scientific reliability, with overall platform performance determined by the median values of the scores across the 17 responses.

The analysis revealed that the most frequent key phrases utilized by consumers were “chest pain in children”, “causes of chest pain”, and “pediatric cardiology”. The remaining core terms forming the study's data pool included: “chest pain left side”, “chest pain right side”, “tachycardia”, “chest pain differential diagnosis”, “pericarditis”, “angina”, “chest pain in kids”, “myocarditis”, “sharp pain in chest”, “pediatric ekg”, “heart attack symptoms”, “heartburn chest pain”, “chest pain when coughing”, and “precordial catch syndrome”. The investigation focused specifically on the datasets associated with these 17 filtered search terms. Geographical distribution data extracted via Google Trends indicated that the highest global search volume for the medical term “pediatric chest pain” originated from the United States, Qatar, and the United Kingdom, respectively.

The identified search queries were simultaneously fed into Perplexity, ChatGPT, and Google Gemini. The linguistic readability performance of the textual outputs was calculated based on Calculator 1, Calculator 2, and the arithmetic mean scores of these two independent platforms. The final readability levels were comparatively evaluated against the sixth-grade reading level criterion, which is recognized as the gold standard for public health materials by the AMA and NIH (Tables 1–3).

**Table 1:** Readability Scores (Calculator-1).

CALCULATOR Statistics	ChatGPT	Gemini	Perplexity	ChatGPT C6thGRL (P)** †	Gemini C6thGRL (P)** †	Perplexity C6thGRL (p)** †	Between ChatGPT and Gemini (p)††	Between ChatGPT and Perplexity (p)††	Between Perplexity and Gemini (p)††
**FRES**	34.59 ± 9.59 (13.00–48.00)	37.41 ± 10.53 (22.00–57.00)	24.94 ± 8.94 (7.00–39.00)	< .001	< .001	< .001	0.717	**0.005**	**0.002**
**GFOG**	14.41 ± 2.22 (10.90–20.00)	13.51 ± 1.95 (10.00–17.70)	15.71 ± 2.48 (11.30–19.40)	< .001	< .001	< .001	0.480	0.113	**0.014**
**FKGL**	11.35 ± 1.62 (9.35–15.52)	11.26 ± 1.41 (8.77–14.51)	13.23 ± 1.47 (11.00–16.26)	< .001	< .001	< .001	0.850	**0.001**	**0.001**
**CLI**	15.32 ± 1.85 (12.24–18.87)	14.70 ± 2.31 (11.01–19.06)	16.89 ± 1.40 (14.12–19.11)	< .001	< .001	< .001	0.428	**0.014**	**0.003**
**SMOG**	9.81 ± 1.52 (8.21–14.11)	9.75 ± 1.02 (8.37–12.69)	11.17 ± 1.26 (9.00–13.31)	< .001	< .001	< .001	0.796	**0.004**	**0.001**
**ARI**	12.62 ± 1.92 (9.57–17.18)	12.30 ± 1.96 (8.61–15.53)	14.29 ± 1.48 (11.33–16.70)	< .001	< .001	< .001	0.796	**0.006**	**0.006**

Calculator 1: https://readabilityformulas.com/free-readability-formula-tests.php.

Calculator 2: https://www.online-utility.org/english/readability_test_and_improve.jsp.

Abbreviations: Flesch reading ease score (FRES), Gunning FOG (GFOG), Flesch-Kincaid Grade Level (FKGL), Simple Measure of Gobbledygook (SMOG), Coleman-Liau Index (CLI), Automated Readability Index (ARI) and Linsear Write (LW).

**: C6thGRL**(p):** Comparison of the responses according to 6th grade reading level **(p)**.

†: Wilcoxon test.

††: Chi-Square test for categorical variables and Mann-Whitney U test for continuous variables.

p *values* in *bold* are *statistically significant.*

**Table 2:** Readability Scores (Calculator-2).

CALCULATOR Statistics	ChatGPT	Gemini	Perplexity	ChatGPT C6thGRL (P)** †	Gemini C6thGRL (P)** †	Perplexity C6thGRL (p)** †	Between ChatGPT and Gemini (p)††	Between ChatGPT and Perplexity (p)††	Between Perplexity and Gemini (p)††
**FRES**	30.17 ± 9.09 (10.99–43.04)	32.65 ± 12.06 (12.25–57.96)	19.49 ± 10.39 (2.96–40.74)	< .001	< .001	< .001	0.986	**0.008**	**0.004**
**GFOG**	14.61 ± 2.24 (11.46–20.00)	14.76 ± 1.83 (11.72–17.71)	16.99 ± 2.63 (12.71–22.78)	< .001	< .001	< .001	0.877	**0.008**	**0.009**
**FKGL**	12.29 ± 1.88 (9.70–16.70)	12.69 ± 1.68 (8.65–15.05)	15.03 ± 2.12 (11.09–18.86)	< .001	< .001	< .001	0.301	**0.001**	**0.002**
**CLI**	14.66 ± 1.99 (10.57–18.37)	14.83 ± 2.03 (11.09–18.28)	16.74 ± 1.53 (14.04–18.83)	< .001	< .001	< .001	0.582	**0.003**	**0.008**
**SMOG**	13.17 ± 1.71 (9.20–16.96)	13.66 ± 1.13 (11.02–15.08)	15.52 ± 2.13 (10.90–19.70)	< .001	< .001	< .001	0.235	**0.002**	**0.004**
**ARI**	11.78 ± 2.11 (8.80–16.48)	12.60 ± 1.64 (8.78–14.89)	15.10 ± 2.54 (9.46–19.80)	< .001	< .001	< .001	0.109	**0.001**	**0.002**

Calculator 1: https://readabilityformulas.com/free-readability-formula-tests.php.

Calculator 2: https://www.online-utility.org/english/readability_test_and_improve.jsp.

Abbreviations: Flesch reading ease score (FRES), Gunning FOG (GFOG), Flesch-Kincaid Grade Level (FKGL), Simple Measure of Gobbledygook (SMOG), Coleman-Liau Index (CLI), Automated Readability Index (ARI) and Linsear Write (LW).

**: C6thGRL**(p):** Comparison of the responses according to 6th grade reading level **(p)**.

†: Wilcoxon test.

††: Chi-Square test for categorical variables and Mann-Whitney U test for continuous variables.

p *values* in *bold* are *statistically significant.*

**Table 3:** Readability Scores (Average of Calculators).

CALCULATOR Statistics	ChatGPT	Gemini	Perplexity	ChatGPT C6thGRL (P)** †	Gemini C6thGRL (P)** †	Perplexity C6thGRL (p)** †	Between ChatGPT and Gemini (p)††	Between ChatGPT and Perplexity (p)††	Between Perplexity and Gemini (p)††
**FRES**	32.38 ± 8.87 (13.33–43.08)	35.03 ± 11.22 (17.13–57.48)	22.22 ± 9.43 (4.98–39.87)	< .001	< .001	< .001	0.959	**0.007**	**0.002**
**GFOG**	14.51 ± 2.09 (11.32–20.00)	14.14 ± 1.81 (11.33–17.71)	16.35 ± 2.29 (12.07–19.82)	< .001	< .001	< .001	0.863	0.017	**0.007**
**FKGL**	11.82 ± 1.57 (10.18–16.11)	11.97 ± 1.47 (8.71–14.71)	14.13 ± 1.60 (11.24–17.05)	< .001	< .001	< .001	0.380	**<0.001**	**0.001**
**CLI**	14.99 ± 1.76 (12.25–18.50)	14.77 ± 2.16 (11.05–18.67)	16.81 ± 1.34 (14.51–18.88)	< .001	< .001	< .001	0.877	**0.003**	**0.005**
**SMOG**	11.49 ± 1.44 (9.25–15.54)	11.71 ± 0.94 (9.93–13.86)	13.34 ± 1.40 (10.90–15.51)	< .001	< .001	< .001	0.310	**0.001**	**0.001**
**ARI**	12.20 ± 1.71 (10.52–16.83)	12.45 ± 1.71 (9.14–15.21)	14.69 ± 1.69 (11.73–17.03)	< .001	< .001	< .001	0.418	**<0.001**	**0.002**

Calculator 1: https://readabilityformulas.com/free-readability-formula-tests.php.

Calculator 2: https://www.online-utility.org/english/readability_test_and_improve.jsp.

Abbreviations: Flesch reading ease score (FRES), Gunning FOG (GFOG), Flesch-Kincaid Grade Level (FKGL), Simple Measure of Gobbledygook (SMOG), Coleman-Liau Index (CLI), Automated Readability Index (ARI) and Linsear Write (LW).

**: C6thGRL**(p):** Comparison of the responses according to 6th grade reading level **(p)**.

†: Wilcoxon test.

††: Chi-Square test for categorical variables and Mann-Whitney U test for continuous variables.

p *values* in *bold* are *statistically significant.*

### Readability findings for calculator 1 data

Linguistic evaluations of the responses to the 17 unique query combinations conducted via Calculator 1 (readabilityformulas.com) demonstrated a statistically highly significant deviation between the median scores of all three AI models and the target 6th-grade level recommended by the NIH/AMA (p < 0.001). Post-hoc pairwise comparisons between the models showed significant performance differences between ChatGPT and Perplexity across the CLI (p = 0.014), FRES (p = 0.005), SMOG (p = 0.004), FKGL (p = 0.001), and ARI (p = 0.006) indices. Similarly, comparing Gemini and Perplexity outputs revealed statistically significant variances across all evaluated parameters: FRES (p = 0.002), FKGL (p = 0.001), CLI (p = 0.003), GFOG (p = 0.014), SMOG (p = 0.001), and ARI (p = 0.006). Conversely, no statistically significant differences were observed in any readability formula when comparing the textual content generated by Gemini and ChatGPT (Table 1).

### Readability findings for calculator 2 data

Linguistic complexity analyses of the text files generated for the 17 independent queries, evaluated through the digital interface of Calculator 2 (online-utility.org), showed a statistically highly significant difference between the median scores of all three AI chatbots and the reference 6th-grade comprehension threshold (p < 0.001). Post-hoc pairwise comparison matrices indicated significant performance variations between ChatGPT and Perplexity across all indices: SMOG (p = 0.001), GFOG (p = 0.008), FRES (p = 0.008), CLI (p = 0.003), FKGL (p = 0.001), and ARI (p = 0.002). Similarly, comparing data from Gemini and Perplexity showed significant differences across all metrics: FRES (p = 0.004), SMOG (p = 0.004), CLI (p = 0.008), FKGL (p = 0.002), GFOG (p = 0.009), and ARI (p = 0.002). In contrast, comparisons between the text responses of Gemini and ChatGPT revealed no statistically significant variations across any of the tested readability formulas (Table 2).

### Text readability analysis based on the mean of calculators 1 and 2

Readability analysis utilizing the averaged scores of Calculators 1 and 2 for the 17 distinct responses demonstrated that the median readability scores of all three AI chatbots differed significantly from the sixth-grade benchmark (p < 0.001). Pairwise post-hoc comparisons indicated significant performance differences between ChatGPT and Perplexity across the FKGL (p < 0.001), SMOG (p = 0.001), FRES (p = 0.007), CLI (p = 0.003), and ARI (p < 0.001) indices. Similarly, comparing Gemini and Perplexity datasets demonstrated significant divergence across all metrics: FRES (p = 0.002), SMOG (p = 0.001), FKGL (p = 0.001), GFOG (p = 0.007), CLI (p = 0.005), and ARI (p = 0.002). No statistically significant divergence was found between Gemini and ChatGPT outputs for any readability metrics (Table 2). Statistical interpretation confirmed that ChatGPT and Gemini produced text with the lowest linguistic complexity and highest accessibility, whereas Perplexity generated responses with greater linguistic density and higher reading difficulty (Table 3).

### Content quality and scientific reliability findings

Table 4 presents detailed distribution profiles for the EQIP, GQS, JAMA, and mDISCERN scores of the responses generated by the AI chatbots. Comparing the overall performance of the three models revealed statistically highly significant differences across all evaluated criteria, including JAMA, mDISCERN, GQS, and EQIP (p < 0.05). Post-hoc pairwise comparison matrices indicated that Perplexity achieved significantly higher performance scores than ChatGPT across all metrics: EQIP (p = 0.003), GQS (p = 0.002), mDISCERN (p = 0.005), and JAMA (p < 0.001). A similar trend was observed when comparing Perplexity to Gemini, with Perplexity demonstrating superior analytical performance for EQIP (p < 0.001), JAMA (p < 0.001), GQS (p = 0.001) and mDISCERN (p = 0.001). Pairwise comparisons between Gemini and ChatGPT revealed a statistically significant difference only for the JAMA score (p < 0.001).

**Table 4:** Comparison of quality and reliability ratings for the responses from AI Chatbots.

	ChatGPT vs Perplexity			ChatGPT vs Gemini			Perplexity vs Gemini		
	ChatGPT	Perplexity	P	ChatGPT	Gemini	P	Perplexity	Gemini	P
**GQS, n (%)**			**0 .002**			.841			**0.001**
**1-point**	2 (11.8)	0 (0%)		2 (11.8)			0 (0%)	0 (0%)	
**2-point**	4 (23.5)	1 (5.9)		4 (23.5)			1 (5.9)	5 (29.4)	
**3-point**	6 (35.3)	2 (11.8)		6 (35.3)			2 (11.8)	8 (47.1)	
**4-point**	5 (29.4)	14 (82.4)		5 (29.4)			14 (82.4)	4 (23.5)	
**5-point**	0 (0%)	0 (0%)		0 (0%)			0 (0%)	0 (0%)	
**JAMA, n (%)**			**< .001**			**< .001**			**< .001**
**0-point**	8 (47.1)	0 (0%)		8 (47.1)			0 (0%)	1 (5.9)	
**1-point**	9 (52.9)	0 (0%)		9 (52.9)			0 (0%)	11 (64.7)	
**2-point**	0 (0%)	6 (35.3)		0 (0%)			6 (35.3)	5 (29.4)	
**3-point**	0 (0%)	11 (64.7)		0 (0%)			11 (64.7)	0 (0%)	
**4-point**	0 (0%)	0 (0%)		0 (0%)			0 (0%)	0 (0%)	
**m DISCERN, n (%)**			**0.005**			.369			**0.001**
**1-point**	0 (0%)	0 (0%)		0 (0%)			0 (0%)	0 (0%)	
**2-point**	9 (52.9)	2 (11.8)		9 (52.9)			2 (11.8)	12 (70.6)	
**3-point**	6 (35.3)	7 (41.2)		6 (35.3)			7 (41.2)	3 (17.6)	
**4-point**	2 (11.8)	8 (47.1)		2 (11.8)			8 (47.1)	2 (11.8)	
**5-point**	0 (0%)	0 (0%)		0 (0%)	0 (0%)		0 (0%)	0 (0%)	
**EQIP, n(%)**			**0.003**			0.311			**<.001**
**Serious problems with qood quality**	7 (41.2)	0 (0%)		7 (41.2)			0 (0%)	10 (58.8)	
**Good quality with minor problems**	10 (58.8)	17 (100.0)		10 (58.8)			17 (100.0)	7 (41.2)	
**Well written**	0 (0%)	0 (0%)		0 (0%)			0 (0%)	0 (0%)	

Data: median (minimum–maximum).

Statistical differences across the three artificial intelligence platforms were evaluated via the Kruskal–Wallis test, with subsequent post-hoc pairwise matrices executed utilizing the Mann–Whitney U test integrated with a Bonferroni correction where applicable.

Bold font: statistically difference.

These analyses demonstrated that Perplexity achieved significantly higher scores across all evaluation tools compared to both ChatGPT and Gemini. Furthermore, Gemini generated higher scores than ChatGPT specifically regarding the JAMA criteria. Inter-rater agreement analysis between the independent evaluators showed strong consistency across all metrics, ranging from good to excellent. The Intraclass Correlation Coefficient (ICC) values were calculated as 0.748 for mDISCERN, 0.891 for JAMA, 0.812 for GQS, and 0.996 for EQIP.

### Correlation analysis

Correlation testing revealed a strong, positive, and statistically significant relationship among the metrics evaluating reliability and content quality. This finding indicates that quality and reliability parameters are methodologically complementary. Furthermore, significant correlation coefficients were identified among the different readability indices, indicating that text complexity measures yield consistent results across various formulas (Table 5).

**Table 5:** Correlation analysis between variables.

Variables	ARI	Flesch Reading Ease	Gunning Fog	FKGL	Coleman–Liau	SMOG	DISCERN	JAMA	GQS	EQIP
**ARI**	1	−0.796**	0.748**	0.936**	0.828**	0.859**	0.238	0.476**	0.418**	0.563**
**Flesch Reading Ease**	−0.796**	1	−0.871**	−0.898**	−0.913**	−0.740**	−0.228	−0.405**	−0.416**	−0.537**
**Gunning Fog**	0.748**	−0.871**	1	0.891**	0.700**	0.870**	0.164	0.326*	0.262	0.454**
**FKGL**	0.936**	−0.898**	0.891**	1	0.814**	0.935**	0.236	0.490**	0.415**	0.561**
**Coleman–Liau**	0.828**	−0.913**	0.700**	0.814**	1	0.648**	0.217	0.397**	0.453**	0.560**
**SMOG**	0.859**	−0.740**	0.870**	0.935**	0.648**	1	0.217	0.483**	0.343*	0.511**
**DISCERN**	0.238	−0.228	0.164	0.236	0.217	0.217	1	0.383**	0.195	0.225
**JAMA**	0.476**	−0.405**	0.326*	0.490**	0.397**	0.483**	0.383**	1	0.409**	0.448**
**GQS**	0.418**	−0.416**	0.262	0.415**	0.453**	0.343*	0.195	0.409**	1	0.399**
**EQIP**	0.563**	−0.537**	0.454**	0.561**	0.560**	0.511**	0.225	0.448**	0.399**	1

ARI: Automated Readability Index; FKGL: Flesch–Kincaid Grade Level; SMOG: Simple Measure of Gobbledygook; DISCERN: Modified DISCERN Score; JAMA: Journal of the American Medical Association Benchmark Score; GQS: Global Quality Scale; EQIP: Ensuring Quality Information for Patients.

* p < 0.05, ** p < 0.001.

### Findings for inter-parameter correlation analysis

The results of the Spearman rank correlation analysis conducted to evaluate the relationships between the readability, content quality, and scientific reliability metrics derived from the AI platforms are presented in Table 5. The analysis showed extremely strong and statistically highly significant correlations among the readability indices evaluating linguistic complexity (p < 0.001). Specifically, the FKGL parameter correlated positively with the ARI (r = 0.936) and SMOG (r = 0.935) indices. Conversely, the FRES metric inherently exhibited strong negative correlations with the Coleman-Liau (r=−0.913) and FKGL (r=−0.898) indices. Evaluating the associations among the reliability and quality surveys confirmed that the JAMA score maintained weak-to-moderate positive linear relationships with the EQIP (r = 0.448), GQS (r = 0.409), and mDISCERN (r = 0.383) metrics (p < 0.001).

## Discussion

This research reveals that the textual outputs produced by leading large language models (LLMs) concerning pediatric chest pain symptoms possess a linguistic complexity that far exceeds the critical sixth-grade reading level threshold recommended for patients by global authorities such as the AMA and NIH. Data obtained from pairwise post-hoc comparisons between the models demonstrate that the ChatGPT and Gemini platforms exhibit the most optimized output in terms of textual readability performance and are closest to public health information standards, while the Perplexity model presents structurally more complex and difficult-to-follow text architectures. However, when content quality and scientific reliability parameters were examined, Perplexity was found to achieve statistically the highest performance scores compared to the other chatbots. In a direct comparison between Gemini and ChatGPT, it was determined that analytical quality showed a significant divergence in favor of Gemini only based on the JAMA reliability criteria, while there was no statistically significant difference between the two models in overall quality scores.

The outputs related to pediatric chest pain generated by these AI platforms were subjected to a multifaceted macro-evaluation using the mDISCERN and JAMA instruments for scientific reliability, and the GQS and EQIP instruments for clinical quality and linguistic readability. Our current study, being one of the pioneering researches in the current medical literature that analyzes the responses of the most popular LLMs in the clinical topic of pediatric chest pain in a multidimensional and comparative manner, offers a unique methodological and strategic contribution to the digital health informatics literature.

Health literacy is one of the most critical clinical variables determining individuals’ capacity to understand, process, and make appropriate medical decisions regarding basic health information and services [13]. Current epidemiological data shows that over 43 million individuals in the United States fall below the threshold of insufficient health literacy, and approximately half of the adult population in Canada has a basic literacy level below high school, resulting in significant barriers to daily functional processes [23]. Over a three-year period, medical and pharmacy costs were found to be approximately $143 million higher for those with low or insufficient health literacy compared to those with adequate health literacy [24]. Accordingly, the AMA and NIH recommend that the readability standard of medical information documents prepared for patients be kept at a maximum of the 6th-grade reading level as a methodological necessity [5,6]. Consequently, optimizing health content generated by digital platforms and AI systems to overcome these population-based linguistic barriers is of strategic importance for patient safety and the sustainability of evidence-based medicine norms [8].

Extant research assessing digital health literature on pediatric chest pain remains scarce, structurally highlighting that these online resources operate at excessively high readability levels and lack sufficient clinical quality. A study evaluating 65 websites on Google related to pediatric chest pain showed that the websites were much more difficult to read than the NIH-recommended 6th-grade reading level, and the quality and comprehensibility indicators were “not good” [25]. In another study investigating 86 websites on Google related to heart murmur in children, the authors found that the texts were written at a level higher than the recommended 6th-grade reading level, but were still comprehensible and of good quality [26]. A study examining patient education materials on 50 websites on Google related to acute rheumatic fever in children highlighted that the readability level was at the academic literacy level. The authors expressed concerns that the difficulty in reading online information about pediatric cardiac diseases that can cause serious complications could negatively impact public health [27]. In a study examining multimedia information transfer tools for parents regarding Childhood Heart Failure, research was conducted on Google Play, the Apple App Store, and the Google Search engine, and it was found that there were no applications on this subject, only 17 websites, and the websites received low scores in terms of readability and graphics when evaluated with the Materials Suitability Assessment (SAM) scale. The authors emphasized that there is a severe lack of information for parents who prefer such web-based content to learn about this important subject [28].

Similar to our study methodology, studies conducted using AI chatbots are also noteworthy in the literature. A study investigating the information provided by ChatGPT-4 regarding Kawasaki disease emphasized that the platform has significant limitations in terms of accuracy and leaves out important information when presenting its recommendations about this disease to parents and pediatric interns [29]. In another recent study investigating pediatric health advice, 28 caregiver-style pediatric questions were asked to 4 different AI chatbots: ChatGPT-4o, Gemini 2.5 Pro, Grok-4, and DeepSeek-V2. Grok was found to have the highest DISCERN scores, indicating strong reliability; Gemini had the highest quality scores, indicating strong quality; and DeepSeek had the best readability. However, all chatbots provided outputs above the recommended readability level. The authors emphasized that LLMs provide moderately useful child health information, but improvements in readability, resources, and consistency are needed before they can be used routinely by patients [30]. There are also a considerable number of studies in the literature that have yielded positive results regarding AI outputs. It has been reported that families were satisfied with and easily understood the education sheets created for them by ChatGPT-4 regarding Pediatric Cardiac Catheterization. The authors reported that LLMs can be valuable tools to enrich the complex pre-medical examination interview with healthcare professionals by increasing clinical satisfaction and understanding [31]. In another study evaluating chronic pain in children, the same questions were asked to four generative AI chatbots one year apart. It was reported that the responses to the questions asked after one year showed significant improvements in usability, consistency, and readability, and presented a readability level below that of a 7th grader. The authors emphasized the importance of continuous benchmarking as AI technologies develop and stressed the need to accelerate research to ensure the robust and secure integration of AI into clinical practice [32].

The empirical insights generated by this investigation corroborate existing literary trends regarding the readability, substantive quality, and scientific trustworthiness of AI-driven medical documentation. Although these generative frameworks demonstrate an intrinsic capacity to synthesize exhaustive clinical data, they systematically rely on a highly sophisticated, university-level syntactic architecture that acts as a formidable barrier for cohorts with limited digital health literacy [25–30]. Mirroring historical datasets, our findings confirm that all three evaluated large language models produced textual outputs that uniformly transcended the mandated sixth-grade reading comprehension threshold, highlighting a critical deficiency in public accessibility. Conversely, when parsing the dimensions of clinical precision and source accountability, Perplexity demonstrated markedly superior performance scores—a finding that aligns with contemporary medical informatics literature evaluating search-grounded models [6]. Nevertheless, no singular platform achieved an optimal benchmark capable of seamlessly fulfilling established clinical guidelines. The documented presence of citation anomalies, informational omissions, and structural hallucination risks clearly underscores that AI-generated health data continues to suffer from systemic quality and optimization deficits, rendering these tools entirely unsuited to substitute for professional medical consultations. Consequently, when navigating this complex, heterogeneous, and uncalibrated wave of digital literature within uncontrolled information ecosystems, medical practitioners bear an indispensable clinical responsibility. Clinicians must proactively instruct patients and their families to approach online health data with a critical lens, ensuring that digital assertions are systematically cross-examined rather than accepted as absolute truths.

A key finding of this study is the ‘Readability-Quality Paradox’ observed in AI-generated health information. Our comparative analysis reveals a clear trade-off between textual accessibility and clinical reliability: while search-augmented models like Perplexity offer higher content quality and superior source attribution, they utilize complex academic language that exceeds standard health literacy thresholds. Conversely, models such as ChatGPT and Gemini produce simpler, more readable text for the general public, but achieve lower scores in clinical quality and reference verification. This paradox highlights a practical challenge for digital patient education, as the most accurate AI outputs remain difficult for average readers to comprehend, whereas the most accessible outputs carry lower clinical depth. Future medical AI applications must focus on bridging this gap by delivering high-fidelity clinical evidence within accessible language frameworks.Although our study showed that the texts provided by AI chatbots excelled in readability with ChatGPT-5 and in content quality and reliability with Perplexity due to scientific referencing, no matter how advanced artificial intelligence becomes, it can never replace face-to-face physician examination, one-on-one clinical evaluation, and physician-patient communication; these tools should only be considered for preliminary information purposes, and diagnosis and treatment should be based on direct guidance from expert physicians [33]. Future bibliometric and altmetric in-depth analyses, methodological expansions, and comprehensive research integrated with clinical practice can provide a solid foundation for this area [34].

## Limitations

When evaluating the empirical findings of this research, several methodological limitations stemming from its design must be considered. Firstly, the study is limited to a specific time period, such as June 2026, and only English search queries filtered from the Google Trends database and open-access/free versions of the relevant AI models were used. The fact that query data entries were conducted via local IP addresses and without VPN integration carries the risk of reflecting potential textual variations that regional cloud server configurations might produce. Additionally, search queries were submitted as direct keywords rather than conversational, scenario-based patient prompts. Because LLMs adapt their tone and readability based on prompt framing, future studies comparing direct search terms against expanded conversational questions could provide further insights into prompt-dependent readability variations. Furthermore, the stochastic nature of LLMs and the inability to standardize internal hyperparameters, such as maximum token length and temperature, by the user indicate that the obtained outputs are subject to dynamic variability over time. On the other hand, although validation tools that have proven themselves in the medical literature (mDISCERN, JAMA, GQS, EQIP) were preferred to measure content quality and scientific reliability, the fact that these scales structurally question similar clinical and methodological parameters inevitably led to the detection of a high linear relationship pattern in the correlation analyses. Consequently, long-term designs encompassing more heterogeneous language options, expanded model architectures, and unique evaluation instruments that test different structural criteria in future academic projects are critical to mapping the reliability and accessibility of the AI-driven medical information ecosystem.

## Strength of the study

Conversely, the most significant scientific contribution and strength of this study to the medical literature is its pioneering research design that goes beyond simply limiting the outputs of AI-based language models in the clinical field of pediatric chest pain to linguistic complexity; it also examines them using multi-layered analytical instruments such as informational accuracy, methodological consistency, referencing quality, and scientific reliability. Unlike similar studies, the simultaneous and synchronized testing of three of the most widely penetrated generative AI architectures globally under a single methodological template clearly reveals the actual potential and clinical limitations of these digital health channels in public health communication. This multi-dimensional, comparative, and calibrated macro approach produces a strategic and evidence-based database that can guide the optimization and transformation of patient-centered information systems in both active clinical management processes and future medical AI integrations.

## Conclusion

The multidimensional data obtained from this research offer critical implications for the current role of generative AI systems in the child health information ecosystem and the linguistic barriers they may pose to public health. All three LLMs evaluated exceeded the “grade 6 reading level” target set by international health authorities (AMA/NIH) for patient education in a sensitive clinical scenario such as pediatric chest pain; their texts were mostly structured with a complex, university-level language template. A significant “readability-quality paradox” was identified among the models; while the ChatGPT and Gemini platforms produced linguistically simpler and more accessible outputs, Perplexity demonstrated significantly superior and optimized performance in terms of clinical content quality, publication ethics principles, and source referencing reliability and quality. However, Perplexity's documents, which utilize internet-based search engine optimization, exhibited the highest linguistic complexity and consequently the most difficult readability profile to track. None of the AI models examined reached a perfect “gold standard” maturity that fully met clinical guidelines and evidence-based medicine norms; inconsistencies in citations and informational gaps identified in the texts confirmed that these technologies still suffer from significant accuracy and reliability issues in routine patient information practices. Consequently, within the current digital information architecture, AI tools are far from replacing a professional pediatric consultation, comprehensive history-taking, or a physical examination. Therefore, it is a vital clinical responsibility for clinicians to proactively guide parents to critically examine data obtained from digital sources regarding pediatric chest pain, rather than accepting it as absolute truth, and to seek direct professional medical help.

## Supporting information

S1 File Supplementary Data. Raw dataset used for analysis.
(PDF)
