# Supplementary material for: The readability and quality paradox: Comparing ChatGPT, Gemini, and Perplexity outputs on pediatric chest pain queries
Source: PLoS One. 2026 Sep 25;21(9):e0359170. doi: 10.1371/journal.pone.0359170 (PMC13614552; doi:10.1371/journal.pone.0359170)
Supplement: Supplementary file 1 [file pone.0359170.s001.pdf]

```
 GET
  FILE='C:\Users\ILHN\Desktop\pediatric.sav'.
DATASET NAME DataSet1 WINDOW=FRONT.
SORT CASES  BY grup.
SPLIT FILE LAYERED BY grup.
COMPUTE arort=(arcal1 + arcal2) / 2.
EXECUTE.
COMPUTE fleschreadingeaseort=
(fleschreadingeasecal1+ fleschreadingeasecal2) / 2.
EXECUTE.
COMPUTE gfogort=(gfogcal1 + gfogcal2) / 2.
EXECUTE.
COMPUTE fleshgradelevelort=(fleshgradelevelcal1+ fleshgradelevelcal2) / 2.
EXECUTE.
COMPUTE colemanort=(colemanliaucal1+ colemanliaucal2) / 2.
EXECUTE.
COMPUTE smogort=(smogcal1 + smogcal2) / 2.
EXECUTE.
FREQUENCIES VARIABLES=arcal1 fleschreadingeasecal1gfogcal1 fleshgradeleve
lcal1 colemanliaucal1smogcal1 gfogcal2 colemanliaucal2fleshgradelevelcal2
 arcal2 smogcal2 fleschreadingeasecal2arort fleschreadingeaseortgfogort
 fleshgradelevelortcolemanort
    smogort
  /STATISTICS=STDDEV MINIMUM MAXIMUM MEAN MEDIAN
  /ORDER=ANALYSIS.
```

Frequencies

[DataSet1] C:\Users\ILHN\Desktop\pediatric.sav

Statistics

grup			arcal1	fleschreadingeasecal1	gfogcal1	fleshgradelevelcal1	colemanliaucal1
chatgpt	N	Valid	17	17	17	17	17
		Missing	0	0	0	0	0
	Mean		12,6206	34,5882	14,4118	11,3471	15,3235
	Median		12,1000	37,0000	13,9000	10,8200	14,9000
	Std. Deviation		1,91629	9,58553	2,21554	1,61820	1,84860
	Minimum		9,57	13,00	10,90	9,35	12,24
	Maximum		17,18	48,00	20,00	15,52	18,87
gemini	N	Valid	17	17	17	17	17
		Missing	0	0	0	0	0
	Mean		12,3029	37,4118	13,5118	11,2624	14,7041
	Median		12,4500	37,0000	13,8000	11,2200	14,6500
	Std. Deviation		1,95634	10,5301	1,95252	1,40861	2,31070
	Minimum		8,61	22,00	10,00	8,77	11,01
	Maximum		15,53	57,00	17,70	14,51	19,06
perplexity	N	Valid	17	17	17	17	17
		Missing	0	0	0	0	0
	Mean		14,2882	24,9412	15,7118	13,2271	16,8924
	Median		14,4500	25,0000	16,0000	13,4500	16,9700
	Std. Deviation		1,48001	8,94057	2,48014	1,47441	1,39531
	Minimum		11,33	7,00	11,30	11,00	14,12
	Maximum		16,70	39,00	19,40	16,26	19,11

Statistics

grup			smogcal1	gfogcal2	colemanliaucal2	fleshgradelevelcal2	arcal2
chatgpt	N	Valid	17	17	17	17	17
		Missing	0	0	0	0	0
	Mean		9,8094	14,6094	14,6629	12,2912	11,7788
	Median		9,4700	14,4300	14,7100	11,7800	10,7600
	Std. Deviation		1,52444	2,24268	1,99233	1,87861	2,10623
	Minimum		8,21	11,46	10,57	9,70	8,80
	Maximum		14,11	20,00	18,37	16,70	16,48
gemini	N	Valid	17	17	17	17	17
		Missing	0	0	0	0	0
	Mean		9,7529	14,7594	14,8306	12,6859	12,5994
	Median		9,4300	14,3100	14,9400	12,9300	13,0600
	Std. Deviation		1,02123	1,83220	2,02586	1,68228	1,64001
	Minimum		8,37	11,72	11,09	8,65	8,78
	Maximum		12,69	17,71	18,28	15,05	14,89
perplexity	N	Valid	17	17	17	17	17
		Missing	0	0	0	0	0
	Mean		11,1659	16,9918	16,7353	15,0306	15,0971
	Median		10,9100	17,1000	17,0100	15,3300	15,4400
	Std. Deviation		1,25807	2,63440	1,53046	2,12352	2,54318
	Minimum		9,00	12,71	14,04	11,09	9,46
	Maximum		13,31	22,78	18,83	18,86	19,80

Statistics

grup			smogcal2	fleschreadingeasecal2	arort	fleschreadingeaseort	gfogort
chatgpt	N	Valid	17	17	17	17	17
		Missing	0	0	0	0	0
	Mean		13,1729	30,1671	12,1997	32,3776	14,5106
	Median		13,0300	30,7000	11,8500	34,6600	13,8900
	Std. Deviation		1,70583	9,09244	1,71298	8,87288	2,09272
	Minimum		9,20	10,99	10,52	13,33	11,32
	Maximum		16,96	43,04	16,83	43,08	20,00
gemini	N	Valid	17	17	17	17	17
		Missing	0	0	0	0	0
	Mean		13,6618	32,6524	12,4512	35,0321	14,1356
	Median		13,5700	29,2700	12,1000	33,1350	14,0750
	Std. Deviation		1,13141	12,0631	1,70784	11,2217	1,81049
	Minimum		11,02	12,25	9,14	17,13	11,33
	Maximum		15,08	57,96	15,21	57,48	17,70
perplexity	N	Valid	17	17	17	17	17
		Missing	0	0	0	0	0
	Mean		15,5206	19,4929	14,6926	22,2171	16,3518
	Median		15,8100	19,4700	15,0300	21,7500	16,7550
	Std. Deviation		2,12622	10,3865	1,68927	9,43424	2,28926
	Minimum		10,90	2,96	11,73	4,98	12,07
	Maximum		19,70	40,74	17,03	39,87	19,82

Statistics

grup			fleshgradelevelort	colemanort	smogort
chatgpt	N	Valid	17	17	17
		Missing	0	0	0
	Mean		11,8191	14,9932	11,4912
	Median		11,2200	14,6850	11,2900
	Std. Deviation		1,57350	1,76145	1,43716
	Minimum		10,18	12,26	9,24
	Maximum		16,11	18,50	15,54
gemini	N	Valid	17	17	17
		Missing	0	0	0
	Mean		11,9741	14,7674	11,7074
	Median		11,9600	14,7000	11,6650
	Std. Deviation		1,47207	2,15525	,93907
	Minimum		8,71	11,05	9,93
	Maximum		14,71	18,67	13,86
perplexity	N	Valid	17	17	17
		Missing	0	0	0
	Mean		14,1288	16,8138	13,3432
	Median		14,3450	16,9500	13,6300
	Std. Deviation		1,59719	1,33570	1,39545
	Minimum		11,24	14,51	10,90
	Maximum		17,05	18,88	15,51

NPar Tests

Kruskal-Wallis Test

Test Statistics[a,b]

	arcal1	fleschreadingeasecal1	gfogcal1	fleshgradelevelcal1	colemanliaucal1	smogcal1	gfogcal2
Chi-Square	10,186	11,743	6,049	15,198	10,267	12,514	9,363
df	2	2	2	2	2	2	2
Asymp. Sig.	,006	,003	,049	,001	,006	,002	,009

Test Statistics[a,b]

	colemanliaucal2	fleshgradelevelcal2	arcal2	smogcal2	fleschreadingeasecal2	arort	fleschreadingeaseort
Chi-Square	10,963	14,376	15,745	12,897	10,483	15,474	11,157
df	2	2	2	2	2	2	2
Asymp. Sig.	,004	,001	,000	,002	,005	,000	,004

Test Statistics[a,b]

	gfogort	fleshgradelevelort	colemanort	smogort
Chi-Square	8,738	16,243	11,072	15,198
df	2	2	2	2
Asymp. Sig.	,013	,000	,004	,001

a. Kruskal Wallis Test

b. Grouping Variable: grup

```
 NPAR TESTS
  /M-
W= arcal1 fleschreadingeasecal1gfogcal1 fleshgradelevelcal1colemanliauca
l1 smogcal1 gfogcal2 colemanliaucal2fleshgradelevelcal2arcal2 smogcal2 f
leschreadingeasecal2arort fleschreadingeaseortgfogort fleshgradelevelort
 colemanort smogort BY
    grup(1 3)
  /MISSING ANALYSIS.
```

NPar Tests

Mann-Whitney Test

Ranks

	grup	N	Mean Rank	Sum of Ranks
arcal1	chatgpt	17	12,79	217,50
	perplexity	17	22,21	377,50
	Total	34		
fleschreadingeasecal1	chatgpt	17	22,32	379,50
	perplexity	17	12,68	215,50
	Total	34		
gfogcal1	chatgpt	17	14,79	251,50
	perplexity	17	20,21	343,50
	Total	34		
fleshgradelevelcal1	chatgpt	17	11,76	200,00
	perplexity	17	23,24	395,00
	Total	34		
colemanliaucal1	chatgpt	17	13,32	226,50
	perplexity	17	21,68	368,50
	Total	34		
smogcal1	chatgpt	17	12,59	214,00
	perplexity	17	22,41	381,00
	Total	34		
gfogcal2	chatgpt	17	12,94	220,00
	perplexity	17	22,06	375,00
	Total	34		

Ranks

	grup	N	Mean Rank	Sum of Ranks
colemanliaucal2	chatgpt	17	12,41	211,00
	perplexity	17	22,59	384,00
	Total	34		
fleshgradelevelcal2	chatgpt	17	11,88	202,00
	perplexity	17	23,12	393,00
	Total	34		
arcal2	chatgpt	17	11,71	199,00
	perplexity	17	23,29	396,00
	Total	34		
smogcal2	chatgpt	17	12,21	207,50
	perplexity	17	22,79	387,50
	Total	34		
fleschreadingeasecal2	chatgpt	17	22,06	375,00
	perplexity	17	12,94	220,00
	Total	34		
arort	chatgpt	17	11,47	195,00
	perplexity	17	23,53	400,00
	Total	34		
fleschreadingeaseort	chatgpt	17	22,12	376,00
	perplexity	17	12,88	219,00
	Total	34		
gfogort	chatgpt	17	13,44	228,50
	perplexity	17	21,56	366,50
	Total	34		
fleshgradelevelort	chatgpt	17	11,47	195,00
	perplexity	17	23,53	400,00
	Total	34		
colemanort	chatgpt	17	12,47	212,00
	perplexity	17	22,53	383,00
	Total	34		
smogort	chatgpt	17	11,82	201,00
	perplexity	17	23,18	394,00
	Total	34		

Test Statistics[a]

	arcal1	fleschreadingeasecal1	gfogcal1	fleshgradelevelcal1	colemanliaucal1	smogcal1
Mann-Whitney U	64,500	62,500	98,500	47,000	73,500	61,000
Wilcoxon W	217,500	215,500	251,500	200,000	226,500	214,000
Z	-2,756	-2,830	-1,585	-3,358	-2,446	-2,876
Asymp. Sig. (2-tailed)	,006	,005	,113	,001	,014	,004
Exact Sig. [2*(1-tailed Sig.)]	,005[b]	,004[b]	,114[b]	,000[b]	,013[b]	,003[b]

Test Statistics[a]

	gfogcal2	colemanliaucal2	fleshgradelevelcal2	arcal2	smogcal2	fleschreadingeasecal2
Mann-Whitney U	67,000	58,000	49,000	46,000	54,500	67,000
Wilcoxon W	220,000	211,000	202,000	199,000	207,500	220,000
Z	-2,670	-2,979	-3,289	-3,393	-3,100	-2,669
Asymp. Sig. (2-tailed)	,008	,003	,001	,001	,002	,008
Exact Sig. [2*(1-tailed Sig.)]	,007[b]	,002[b]	,001[b]	,000[b]	,001[b]	,007[b]

Test Statistics[a]

	arort	fleschreadingeaseort	gfogort	fleshgradelevelort	colemanort	smogort
Mann-Whitney U	42,000	66,000	75,500	42,000	59,000	48,000
Wilcoxon W	195,000	219,000	228,500	195,000	212,000	201,000
Z	-3,530	-2,704	-2,377	-3,530	-2,945	-3,324
Asymp. Sig. (2-tailed)	,000	,007	,017	,000	,003	,001
Exact Sig. [2*(1-tailed Sig.)]	,000[b]	,006[b]	,016[b]	,000[b]	,003[b]	,001[b]

a. Grouping Variable: grup

b. Not corrected for ties.

```
 NPAR TESTS
  /M-
W= arcal1 fleschreadingeasecal1gfogcal1 fleshgradelevelcal1colemanliauca
l1 smogcal1 gfogcal2 colemanliaucal2fleshgradelevelcal2arcal2 smogcal2 f
leschreadingeasecal2arort fleschreadingeaseortgfogort fleshgradelevelort
 colemanort smogort BY
   grup(1 2)
  /MISSING ANALYSIS.
```

NPar Tests

Mann-Whitney Test

Ranks

	grup	N	Mean Rank	Sum of Ranks
arcal1	chatgpt	17	17,94	305,00
	gemini	17	17,06	290,00
	Total	34		
fleschreadingeasecal1	chatgpt	17	16,88	287,00
	gemini	17	18,12	308,00
	Total	34		
gfogcal1	chatgpt	17	18,71	318,00
	gemini	17	16,29	277,00
	Total	34		
fleshgradelevelcal1	chatgpt	17	17,18	292,00
	gemini	17	17,82	303,00
	Total	34		
colemanliaucal1	chatgpt	17	18,85	320,50
	gemini	17	16,15	274,50
	Total	34		
smogcal1	chatgpt	17	17,06	290,00
	gemini	17	17,94	305,00
	Total	34		
gfogcal2	chatgpt	17	17,24	293,00
	gemini	17	17,76	302,00
	Total	34		
colemanliaucal2	chatgpt	17	16,56	281,50
	gemini	17	18,44	313,50
	Total	34		
fleshgradelevelcal2	chatgpt	17	15,74	267,50
	gemini	17	19,26	327,50
	Total	34		
arcal2	chatgpt	17	14,76	251,00
	gemini	17	20,24	344,00
	Total	34		
smogcal2	chatgpt	17	15,47	263,00
	gemini	17	19,53	332,00
	Total	34		
fleschreadingeasecal2	chatgpt	17	17,53	298,00
	gemini	17	17,47	297,00
	Total	34		
arort	chatgpt	17	16,12	274,00
	gemini	17	18,88	321,00
	Total	34		
fleschreadingeaseort	chatgpt	17	17,41	296,00
	gemini	17	17,59	299,00
	Total	34		

Ranks

	grup	N	Mean Rank	Sum of Ranks
gfogort	chatgpt	17	17,79	302,50
	gemini	17	17,21	292,50
	Total	34		
fleshgradelevelort	chatgpt	17	16,00	272,00
	gemini	17	19,00	323,00
	Total	34		
colemanort	chatgpt	17	17,76	302,00
	gemini	17	17,24	293,00
	Total	34		
smogort	chatgpt	17	15,76	268,00
	gemini	17	19,24	327,00
	Total	34		

Test Statistics[a]

	arcal1	fleschreadingeasecal1	gfogcal1	fleshgradelevelcal1	colemanliaucal1	smogcal1
Mann-Whitney U	137,000	134,000	124,000	139,000	121,500	137,000
Wilcoxon W	290,000	287,000	277,000	292,000	274,500	290,000
Z	-,258	-,362	-,706	-,189	-,792	-,258
Asymp. Sig. (2-tailed)	,796	,717	,480	,850	,428	,796
Exact Sig. [2*(1-tailed Sig.)]	,812[b]	,734[b]	,496[b]	,865[b]	,433[b]	,812[b]

Test Statistics[a]

	gfogcal2	colemanliaucal2	fleshgradelevelcal2	arcal2	smogcal2	fleschreadingeasecal2
Mann-Whitney U	140,000	128,500	114,500	98,000	110,000	144,000
Wilcoxon W	293,000	281,500	267,500	251,000	263,000	297,000
Z	-,155	-,551	-1,033	-1,602	-1,188	-,017
Asymp. Sig. (2-tailed)	,877	,582	,301	,109	,235	,986
Exact Sig. [2*(1-tailed Sig.)]	,892[b]	,586[b]	,306[b]	,114[b]	,245[b]	1,000[b]

Test Statistics[a]

	arort	fleschreadingeaseort	gfogort	fleshgradelevelort	colemanort	smogort
Mann-Whitney U	121,000	143,000	139,500	119,000	140,000	115,000
Wilcoxon W	274,000	296,000	292,500	272,000	293,000	268,000
Z	-,809	-,052	-,172	-,878	-,155	-1,016
Asymp. Sig. (2-tailed)	,418	,959	,863	,380	,877	,310
Exact Sig. [2*(1-tailed Sig.)]	,433[b]	,973[b]	,865[b]	,394[b]	,892[b]	,322[b]

a. Grouping Variable: grup

b. Not corrected for ties.

```
 NPAR TESTS
  /M-
W= arcal1 fleschreadingeasecal1gfogcal1 fleshgradelevelcal1colemanliauca
l1 smogcal1 gfogcal2 colemanliaucal2fleshgradelevelcal2arcal2 smogcal2 f
leschreadingeasecal2arort fleschreadingeaseortgfogort fleshgradelevelort
 colemanort smogort BY
    grup(2 3)
  /MISSING ANALYSIS.
```

NPar Tests

Mann-Whitney Test

Ranks

	grup	N	Mean Rank	Sum of Ranks
arcal1	gemini	17	12,82	218,00
	perplexity	17	22,18	377,00
	Total	34		
fleschreadingeasecal1	gemini	17	22,71	386,00
	perplexity	17	12,29	209,00
	Total	34		
gfogcal1	gemini	17	13,32	226,50
	perplexity	17	21,68	368,50
	Total	34		
fleshgradelevelcal1	gemini	17	11,76	200,00
	perplexity	17	23,24	395,00
	Total	34		
colemanliaucal1	gemini	17	12,47	212,00
	perplexity	17	22,53	383,00
	Total	34		
smogcal1	gemini	17	12,00	204,00
	perplexity	17	23,00	391,00
	Total	34		
gfogcal2	gemini	17	13,06	222,00
	perplexity	17	21,94	373,00
	Total	34		
colemanliaucal2	gemini	17	12,94	220,00
	perplexity	17	22,06	375,00
	Total	34		
fleshgradelevelcal2	gemini	17	12,18	207,00
	perplexity	17	22,82	388,00
	Total	34		
arcal2	gemini	17	12,12	206,00
	perplexity	17	22,88	389,00
	Total	34		
smogcal2	gemini	17	12,53	213,00
	perplexity	17	22,47	382,00
	Total	34		
fleschreadingeasecal2	gemini	17	22,47	382,00
	perplexity	17	12,53	213,00
	Total	34		
arort	gemini	17	12,12	206,00
	perplexity	17	22,88	389,00
	Total	34		
fleschreadingeaseort	gemini	17	22,71	386,00
	perplexity	17	12,29	209,00
	Total	34		

Ranks

	grup	N	Mean Rank	Sum of Ranks
gfogort	gemini	17	12,88	219,00
	perplexity	17	22,12	376,00
	Total	34		
fleshgradelevelort	gemini	17	11,82	201,00
	perplexity	17	23,18	394,00
	Total	34		
colemanort	gemini	17	12,74	216,50
	perplexity	17	22,26	378,50
	Total	34		
smogort	gemini	17	11,88	202,00
	perplexity	17	23,12	393,00
	Total	34		

Test Statistics[a]

	arcal1	fleschreadingeasecal1	gfogcal1	fleshgradelevelcal1	colemanliaucal1	smogcal1
Mann-Whitney U	65,000	56,000	73,500	47,000	59,000	51,000
Wilcoxon W	218,000	209,000	226,500	200,000	212,000	204,000
Z	-2,738	-3,055	-2,446	-3,358	-2,945	-3,221
Asymp. Sig. (2-tailed)	,006	,002	,014	,001	,003	,001
Exact Sig. [2*(1-tailed Sig.)]	,005[b]	,002[b]	,013[b]	,000[b]	,003[b]	,001[b]

Test Statistics[a]

	gfogcal2	colemanliaucal2	fleshgradelevelcal2	arcal2	smogcal2	fleschreadingeasecal2
Mann-Whitney U	69,000	67,000	54,000	53,000	60,000	60,000
Wilcoxon W	222,000	220,000	207,000	206,000	213,000	213,000
Z	-2,601	-2,669	-3,117	-3,152	-2,911	-2,910
Asymp. Sig. (2-tailed)	,009	,008	,002	,002	,004	,004
Exact Sig. [2*(1-tailed Sig.)]	,009[b]	,007[b]	,001[b]	,001[b]	,003[b]	,003[b]

Test Statistics[a]

	arort	fleschreadingeaseort	gfogort	fleshgradelevelort	colemanort	smogort
Mann-Whitney U	53,000	56,000	66,000	48,000	63,500	49,000
Wilcoxon W	206,000	209,000	219,000	201,000	216,500	202,000
Z	-3,152	-3,048	-2,704	-3,324	-2,790	-3,290
Asymp. Sig. (2-tailed)	,002	,002	,007	,001	,005	,001
Exact Sig. [2*(1-tailed Sig.)]	,001[b]	,002[b]	,006[b]	,001[b]	,004[b]	,001[b]

a. Grouping Variable: grup

b. Not corrected for ties.

```
FREQUENCIES VARIABLES=mdiscernnominal jamanominal gqsnominal eqpnominal
  /STATISTICS=STDDEV MINIMUM MAXIMUM MEAN MEDIAN
  /ORDER=ANALYSIS.
```

```
 SORT CASES  BY grup.
SPLIT FILE LAYERED BY grup.
FREQUENCIES VARIABLES=mdiscernnominal jamanominal gqsnominal eqpnominal
  /STATISTICS=STDDEV MINIMUM MAXIMUM MEAN MEDIAN
  /ORDER=ANALYSIS.
```

Frequencies

Frequency Table

mdiscernnominal

grup			Frequency	Percent	Valid Percent	Cumulative Percent
chatgpt	Valid	poor	9	52,9	52,9	52,9
		fair	6	35,3	35,3	88,2
		good	2	11,8	11,8	100,0
		Total	17	100,0	100,0	
gemini	Valid	poor	12	70,6	70,6	70,6
		fair	3	17,6	17,6	88,2
		good	2	11,8	11,8	100,0
		Total	17	100,0	100,0	
perplexity	Valid	poor	2	11,8	11,8	11,8
		fair	7	41,2	41,2	52,9
		good	8	47,1	47,1	100,0
		Total	17	100,0	100,0	

jamanominal

grup			Frequency	Percent	Valid Percent	Cumulative Percent
chatgpt	Valid	insufficient data	17	100,0	100,0	100,0
gemini	Valid	insufficient data	12	70,6	70,6	70,6
		partially sufficient data	5	29,4	29,4	100,0
		Total	17	100,0	100,0	
perplexity	Valid	partially sufficient data	17	100,0	100,0	100,0

gqsnominal

grup			Frequency	Percent	Valid Percent	Cumulative Percent
chatgpt	Valid	low quality	6	35,3	35,3	35,3
		medium quality	6	35,3	35,3	70,6
		high quality	5	29,4	29,4	100,0
		Total	17	100,0	100,0	
gemini	Valid	low quality	5	29,4	29,4	29,4
		medium quality	8	47,1	47,1	76,5
		high quality	4	23,5	23,5	100,0
		Total	17	100,0	100,0	
perplexity	Valid	low quality	1	5,9	5,9	5,9
		medium quality	2	11,8	11,8	17,6
		high quality	14	82,4	82,4	100,0
		Total	17	100,0	100,0	

eqpnominal

grup			Frequency	Percent	Valid Percent	Cumulative Percent
chatgpt	Valid	serious problems with quality	7	41,2	41,2	41,2
		good quality with minor problems	10	58,8	58,8	100,0
		Total	17	100,0	100,0	
gemini	Valid	serious problems with quality	10	58,8	58,8	58,8
		good quality with minor problems	7	41,2	41,2	100,0
		Total	17	100,0	100,0	
perplexity	Valid	good quality with minor problems	17	100,0	100,0	100,0

```
NPAR TESTS
 /K-W=mdiscernnominal jamanominal gqsnominal eqpnominal BY grup(1 3)
```

```
  /MISSING ANALYSIS.
```

NPar Tests

Kruskal-Wallis Test

```
 SPLIT FILE OFF.
NPAR TESTS
  /K-W=mdiscernnominal jamanominal gqsnominal eqpnominal BY grup(1 3)
  /MISSING ANALYSIS.
```

NPar Tests

Kruskal-Wallis Test

Ranks

	grup	N	Mean Rank
mdiscernnominal	chatgpt	17	22,82
	gemini	17	19,38
	perplexity	17	35,79
	Total	51	
jamanominal	chatgpt	17	15,00
	gemini	17	22,50
	perplexity	17	40,50
	Total	51	
gqsnominal	chatgpt	17	21,29
	gemini	17	20,97
	perplexity	17	35,74
	Total	51	
eqpnominal	chatgpt	17	24,00
	gemini	17	19,50
	perplexity	17	34,50
	Total	51	

Test Statistics[a,b]

	mdiscernnominal	jamanominal	gqsnominal	eqpnominal
Chi-Square	13,327	35,893	12,651	13,668
df	2	2	2	2
Asymp. Sig.	,001	,000	,002	,001

a. Kruskal Wallis Test

b. Grouping Variable: grup

```
 NPAR TESTS
  /M-W= mdiscernnominal jamanominal gqsnominal eqpnominal BY grup(1 3)
```

```
/MISSING ANALYSIS.
```

NPar Tests

Mann-Whitney Test

Ranks

	grup	N	Mean Rank	Sum of Ranks
mdiscernnominal	chatgpt	17	13,00	221,00
	perplexity	17	22,00	374,00
	Total	34		
jamanominal	chatgpt	17	9,00	153,00
	perplexity	17	26,00	442,00
	Total	34		
gqsnominal	chatgpt	17	12,82	218,00
	perplexity	17	22,18	377,00
	Total	34		
eqpnominal	chatgpt	17	14,00	238,00
	perplexity	17	21,00	357,00
	Total	34		

Test Statistics[a]

	mdiscernnominal	jamanominal	gqsnominal	eqpnominal
Mann-Whitney U	68,000	,000	65,000	85,000
Wilcoxon W	221,000	153,000	218,000	238,000
Z	-2,800	-5,745	-3,053	-2,925
Asymp. Sig. (2-tailed)	,005	,000	,002	,003
Exact Sig. [2*(1-tailed Sig.)]	,008[b]	,000[b]	,005[b]	,041[b]

a. Grouping Variable: grup

b. Not corrected for ties.

```
NPAR TESTS
 /M-W= mdiscernnominal jamanominal gqsnominal eqpnominal BY grup(1 2)
 /MISSING ANALYSIS.
```

NPar Tests

Mann-Whitney Test

Ranks

	grup	N	Mean Rank	Sum of Ranks
mdiscernnominal	chatgpt	17	18,82	320,00
	gemini	17	16,18	275,00
	Total	34		
jamanominal	chatgpt	17	15,00	255,00
	gemini	17	20,00	340,00
	Total	34		
gqsnominal	chatgpt	17	17,47	297,00
	gemini	17	17,53	298,00
	Total	34		
eqpnominal	chatgpt	17	19,00	323,00
	gemini	17	16,00	272,00
	Total	34		

Test Statistics[a]

	mdiscernnominal	jamanominal	gqsnominal	eqpnominal
Mann-Whitney U	122,000	102,000	144,000	119,000
Wilcoxon W	275,000	255,000	297,000	272,000
Z	-,898	-2,385	-,018	-1,014
Asymp. Sig. (2-tailed)	,369	,017	,985	,311
Exact Sig. [2*(1-tailed Sig.)]	,454[b]	,150[b]	1,000[b]	,394[b]

a. Grouping Variable: grup

b. Not corrected for ties.

```
NPAR TESTS
 /M-W= mdiscernnominal jamanominal gqsnominal eqpnominal BY grup(2 3)
 /MISSING ANALYSIS.
```

NPar Tests

Mann-Whitney Test

Ranks

	grup	N	Mean Rank	Sum of Ranks
mdiscernnominal	gemini	17	12,21	207,50
	perplexity	17	22,79	387,50
	Total	34		
jamanominal	gemini	17	11,50	195,50
	perplexity	17	23,50	399,50
	Total	34		
gqsnominal	gemini	17	12,44	211,50
	perplexity	17	22,56	383,50
	Total	34		
eqpnominal	gemini	17	12,50	212,50
	perplexity	17	22,50	382,50
	Total	34		

Test Statistics[a]

	mdiscernnominal	jamanominal	gqsnominal	eqpnominal
Mann-Whitney U	54,500	42,500	58,500	59,500
Wilcoxon W	207,500	195,500	211,500	212,500
Z	-3,304	-4,243	-3,268	-3,708
Asymp. Sig. (2-tailed)	,001	,000	,001	,000
Exact Sig. [2*(1-tailed Sig.)]	,001[b]	,000[b]	,002[b]	,003[b]

a. Grouping Variable: grup

b. Not corrected for ties.

```
CORRELATIONS
  /VARIABLES=arort fleschreadingeaseort gfogort fleshgradelevelort colemanort smogort mdiscernnominal jamanominal gqsnominal eqpnominal
  /PRINT=TWOTAIL NOSIG
  /MISSING=PAIRWISE.
```

Correlations

Correlations

		arort	fleschreadingeaseort	gfogort	fleshgradelevelort	colemanort
arort	Pearson Correlation	1	-,796**	,748**	,936**	,828**
	Sig. (2-tailed)		,000	,000	,000	,000
	N	51	51	51	51	51
fleschreadingeaseort	Pearson Correlation	-,796**	1	-,871**	-,898**	-,913**
	Sig. (2-tailed)	,000		,000	,000	,000
	N	51	51	51	51	51
gfogort	Pearson Correlation	,748**	-,871**	1	,891**	,700**
	Sig. (2-tailed)	,000	,000		,000	,000
	N	51	51	51	51	51
fleshgradelevelort	Pearson Correlation	,936**	-,898**	,891**	1	,814**
	Sig. (2-tailed)	,000	,000	,000		,000
	N	51	51	51	51	51
colemanort	Pearson Correlation	,828**	-,913**	,700**	,814**	1
	Sig. (2-tailed)	,000	,000	,000	,000	
	N	51	51	51	51	51
smogort	Pearson Correlation	,859**	-,740**	,870**	,935**	,648**
	Sig. (2-tailed)	,000	,000	,000	,000	,000
	N	51	51	51	51	51
mdiscernnominal	Pearson Correlation	,238	-,228	,164	,236	,217
	Sig. (2-tailed)	,093	,108	,249	,095	,127
	N	51	51	51	51	51
jamanominal	Pearson Correlation	,476**	-,405**	,326*	,490**	,397**
	Sig. (2-tailed)	,000	,003	,019	,000	,004
	N	51	51	51	51	51
gqsnominal	Pearson Correlation	,418**	-,416**	,262	,415**	,453**
	Sig. (2-tailed)	,002	,002	,063	,002	,001
	N	51	51	51	51	51
eqpnominal	Pearson Correlation	,563**	-,537**	,454**	,561**	,560**
	Sig. (2-tailed)	,000	,000	,001	,000	,000
	N	51	51	51	51	51

Correlations

		smogort	mdiscernnominal	jamanominal	gqsnominal	eqpnominal
arort	Pearson Correlation	,859**	,238	,476**	,418**	,563**
	Sig. (2-tailed)	,000	,093	,000	,002	,000
	N	51	51	51	51	51
fleschreadingeaseort	Pearson Correlation	-,740**	-,228	-,405**	-,416**	-,537**
	Sig. (2-tailed)	,000	,108	,003	,002	,000
	N	51	51	51	51	51
gfogort	Pearson Correlation	,870**	,164	,326*	,262	,454**
	Sig. (2-tailed)	,000	,249	,019	,063	,001
	N	51	51	51	51	51
fleshgradelevelort	Pearson Correlation	,935**	,236	,490**	,415**	,561**
	Sig. (2-tailed)	,000	,095	,000	,002	,000
	N	51	51	51	51	51
colemanort	Pearson Correlation	,648**	,217	,397**	,453**	,560**
	Sig. (2-tailed)	,000	,127	,004	,001	,000
	N	51	51	51	51	51
smogort	Pearson Correlation	1	,217	,483**	,343*	,511**
	Sig. (2-tailed)		,125	,000	,014	,000
	N	51	51	51	51	51
mdiscernnominal	Pearson Correlation	,217	1	,383**	,195	,225
	Sig. (2-tailed)	,125		,005	,170	,112
	N	51	51	51	51	51
jamanominal	Pearson Correlation	,483**	,383**	1	,409**	,448**
	Sig. (2-tailed)	,000	,005		,003	,001
	N	51	51	51	51	51
gqsnominal	Pearson Correlation	,343*	,195	,409**	1	,399**
	Sig. (2-tailed)	,014	,170	,003		,004
	N	51	51	51	51	51
eqpnominal	Pearson Correlation	,511**	,225	,448**	,399**	1
	Sig. (2-tailed)	,000	,112	,001	,004	
	N	51	51	51	51	51

**. Correlation is significant at the 0.01 level (2-tailed).

*. Correlation is significant at the 0.05 level (2-tailed).

GET

```
  FILE='C:\Users\ILHN\Desktop\pediatri\pediatric.sav'.
DATASET NAME DataSet1 WINDOW=FRONT.
COMPUTE jamaort=(jama1 + jama2) / 2.
EXECUTE.
COMPUTE dscernort=(modifiyediscern1+ modifiyediscern2) / 2.
EXECUTE.
COMPUTE gqsort=(gqs1 + gqs2) / 2.
EXECUTE.
SORT CASES  BY grup.
SPLIT FILE LAYERED BY grup.
DESCRIPTIVES VARIABLES=jamaort dscernort gqsort
  /STATISTICS=MEAN STDDEV MIN MAX.
```

Descriptives

[DataSet1] C:\Users\ILHN\Desktop\pediatri\pediatric.sav

Descriptive Statistics

grup		N	Minimum	Maximum	Mean	Std. Deviation
chatgpt	jamaort	17	,00	1,00	,5294	,51450
	dscernort	17	2,00	4,00	2,5882	,71229
	gqsort	17	1,00	4,00	2,8235	1,01460
	Valid N (listwise)	17				
gemini	jamaort	17	,00	2,00	1,2353	,56230
	dscernort	17	2,00	4,00	2,4118	,71229
	gqsort	17	2,00	4,00	2,9412	,74755
	Valid N (listwise)	17				
perplexity	jamaort	17	2,00	3,00	2,6471	,49259
	dscernort	17	2,00	4,00	3,3529	,70189
	gqsort	17	2,00	4,00	3,7647	,56230
	Valid N (listwise)	17				

```
 FREQUENCIES VARIABLES=jamaort dscernort gqsort
  /ORDER=ANALYSIS.
```

Frequencies

Statistics

grup			jamaort	dscernort	gqsort
chatgpt	N	Valid	17	17	17
		Missing	0	0	0
gemini	N	Valid	17	17	17
		Missing	0	0	0
perplexity	N	Valid	17	17	17
		Missing	0	0	0

Frequency Table

jamaort

grup			Frequency	Percent	Valid Percent	Cumulative Percent
chatgpt	Valid	,00	8	47,1	47,1	47,1
		1,00	9	52,9	52,9	100,0
		Total	17	100,0	100,0	
gemini	Valid	,00	1	5,9	5,9	5,9
		1,00	11	64,7	64,7	70,6
		2,00	5	29,4	29,4	100,0
		Total	17	100,0	100,0	
perplexity	Valid	2,00	6	35,3	35,3	35,3
		3,00	11	64,7	64,7	100,0
		Total	17	100,0	100,0	

dscernort

grup			Frequency	Percent	Valid Percent	Cumulative Percent
chatgpt	Valid	2,00	9	52,9	52,9	52,9
		3,00	6	35,3	35,3	88,2
		4,00	2	11,8	11,8	100,0
		Total	17	100,0	100,0	
gemini	Valid	2,00	12	70,6	70,6	70,6
		3,00	3	17,6	17,6	88,2
		4,00	2	11,8	11,8	100,0
		Total	17	100,0	100,0	
perplexity	Valid	2,00	2	11,8	11,8	11,8
		3,00	7	41,2	41,2	52,9
		4,00	8	47,1	47,1	100,0
		Total	17	100,0	100,0	

gqsort

grup			Frequency	Percent	Valid Percent	Cumulative Percent
chatgpt	Valid	1,00	2	11,8	11,8	11,8
		2,00	4	23,5	23,5	35,3
		3,00	6	35,3	35,3	70,6
		4,00	5	29,4	29,4	100,0
		Total	17	100,0	100,0	
gemini	Valid	2,00	5	29,4	29,4	29,4
		3,00	8	47,1	47,1	76,5
		4,00	4	23,5	23,5	100,0
		Total	17	100,0	100,0	
perplexity	Valid	2,00	1	5,9	5,9	5,9
		3,00	2	11,8	11,8	17,6
		4,00	14	82,4	82,4	100,0
		Total	17	100,0	100,0	

```
NPAR TESTS
 /M-W= jamaort dscernort gqsort BY grup(1 3)
 /MISSING ANALYSIS.
```

NPar Tests

Warnings

There are not enough valid cases to perform the Mann-Whitney Test for jamaort * grup (chatgpt, perplexity) in split file grup=gemini. No statistics are computed.
There are not enough valid cases to perform the Mann-Whitney Test for dscernort * grup (chatgpt, perplexity) in split file grup=gemini. No statistics are computed.
There are not enough valid cases to perform the Mann-Whitney Test for gqsort * grup (chatgpt, perplexity) in split file grup=gemini. No statistics are computed.

Mann-Whitney Test

Ranks

	grup	N	Mean Rank	Sum of Ranks
jamaort	chatgpt	0[a]	,00	,00
	perplexity	17[a]	9,00	153,00
	Total	17		
dscernort	chatgpt	0[a]	,00	,00
	perplexity	17[a]	9,00	153,00
	Total	17		
gqsort	chatgpt	0[a]	,00	,00
	perplexity	17[a]	9,00	153,00
	Total	17		

a. Mann-Whitney Test cannot be performed on empty groups.

```
 SPLIT FILE OFF.
NPAR TESTS
  /M-W= jamaort dscernort gqsort BY grup(1 3)
  /MISSING ANALYSIS.
```

NPar Tests

Mann-Whitney Test

Ranks

	grup	N	Mean Rank	Sum of Ranks
jamaort	chatgpt	17	9,00	153,00
	perplexity	17	26,00	442,00
	Total	34		
dscernort	chatgpt	17	13,00	221,00
	perplexity	17	22,00	374,00
	Total	34		
gqsort	chatgpt	17	12,76	217,00
	perplexity	17	22,24	378,00
	Total	34		

Test Statistics[a]

	jamaort	dscernort	gqsort
Mann-Whitney U	,000	68,000	64,000
Wilcoxon W	153,000	221,000	217,000
Z	-5,161	-2,800	-3,081
Asymp. Sig. (2-tailed)	,000	,005	,002
Exact Sig. [2*(1-tailed Sig.)]	,000[b]	,008[b]	,005[b]

a. Grouping Variable: grup

b. Not corrected for ties.

```
NPAR TESTS
 /M-W= jamaort dscernort gqsort BY grup(1 2)
 /MISSING ANALYSIS.
```

NPar Tests

Mann-Whitney Test

Ranks

	grup	N	Mean Rank	Sum of Ranks
jamaort	chatgpt	17	12,68	215,50
	gemini	17	22,32	379,50
	Total	34		
dscernort	chatgpt	17	18,82	320,00
	gemini	17	16,18	275,00
	Total	34		
gqsort	chatgpt	17	17,18	292,00
	gemini	17	17,82	303,00
	Total	34		

Test Statistics[a]

	jamaort	dscernort	gqsort
Mann-Whitney U	62,500	122,000	139,000
Wilcoxon W	215,500	275,000	292,000
Z	-3,207	-,898	-,200
Asymp. Sig. (2-tailed)	,001	,369	,841
Exact Sig. [2*(1-tailed Sig.)]	,004[b]	,454[b]	,865[b]

a. Grouping Variable: grup

b. Not corrected for ties.

```
NPAR TESTS
 /M-W= jamaort dscernort gqsort BY grup(2 3)
 /MISSING ANALYSIS.
```

NPar Tests

Mann-Whitney Test

Ranks

	grup	N	Mean Rank	Sum of Ranks
jamaort	gemini	17	9,88	168,00
	perplexity	17	25,12	427,00
	Total	34		
dscernort	gemini	17	12,21	207,50
	perplexity	17	22,79	387,50
	Total	34		
gqsort	gemini	17	12,44	211,50
	perplexity	17	22,56	383,50
	Total	34		

Test Statistics[a]

	jamaort	dscernort	gqsort
Mann-Whitney U	15,000	54,500	58,500
Wilcoxon W	168,000	207,500	211,500
Z	-4,704	-3,304	-3,268
Asymp. Sig. (2-tailed)	,000	,001	,001
Exact Sig. [2*(1-tailed Sig.)]	,000[b]	,001[b]	,002[b]

a. Grouping Variable: grup

b. Not corrected for ties.
