# Supplementary material for: The readability and quality paradox: Comparing ChatGPT, Gemini, and Perplexity outputs on pediatric chest pain queries
Source: PLoS One. 2026 Sep 25;21(9):e0359170. doi: 10.1371/journal.pone.0359170 (PMC13614552; doi:10.1371/journal.pone.0359170)
Supplement: Supplementary file 2 [file pone.0359170.s003.docx]

**Dear Editor, Thank you very much for giving us the opportunity to revise our manuscript. I would like to thank the editor and the reviewer for their valuable comments. We have now carefully revised our manuscript according to the comments as indicated below.**

Reviewer #1: This manuscript presents an exceptionally well-designed and highly timely study that addresses a critical gap at the intersection of pediatric care and digital health technology. By systematically examining the 'readability-quality paradox' across modern AI language models, the authors offer invaluable, evidence-based insights into how online medical information reaches patients and caregivers. The methodological framework is admirably rigorous, combining comprehensive search trend analyses with standardized readability formulas and validated clinical quality instruments. Furthermore, the narrative is eloquently constructed, striking a perfect balance between nuanced scientific analysis and actionable clinical implications. Overall, this is an outstanding, pioneering contribution to medical informatics that will undoubtedly serve as a benchmark for future research in digital health literacy and AI-assisted patient communication

1. Typographical and Grammatical Errors (Typo/Formatting Issues): There are several noticeable typos throughout the text that have been overlooked. They must be corrected before publication: "sixthth-grade": It is written as "sixthth" in several places in the abstract and the text. The correct spelling should be "6th-grade" or "sixth-grade".

We sincerely thank the reviewer for pointing out this oversight. We agree with the recommendation. All instances of the typographical error "sixthth-grade" have been meticulously identified and corrected to "sixth-grade" throughout the Abstract, Material and Methods, Results, and Discussion sections. Additionally, the entire manuscript has been thoroughly re-proofread to eliminate any other potential typographical or formatting errors.


2. Criticisms Regarding Methodology: Prompt Design: Were search terms (e.g., "pediatric chest pain") written directly into the AI, or were they presented in a patient/parent question format such as "My child has chest pain, what should I do?" This distinction directly affects readability results. This part needs clarification in the text.

We thank the reviewer for highlighting this important methodological detail. In our study, the search terms identified via Google Trends (e.g., *"chest pain in children"*, *"pediatric chest pain"*) were entered directly into the AI models as exact queries, rather than being rephrased into expanded conversational questions.

This approach was intentionally selected for two reasons:

1. To maintain methodological consistency with the exact top-searched keywords retrieved from the Google Trends database.
2. To avoid introducing prompt-engineering bias (such as requesting the AI to "explain like I am 5" or adopting a specific persona), thereby capturing the models' default, unprompted output behavior when presented with standard search phrases.

We acknowledge that prompt structure directly influences output readability and complexity. To make this fully transparent, we have updated the **Material and Methods** section to explicitly state that direct query terms were used and added a brief reflection on this in the **Limitations** section.

Revised Text:
Methods
To eliminate prompt-engineering bias and evaluate the default baseline outputs of the AI platforms, the filtered search terms extracted from Google Trends were entered directly into the LLMs as verbatim queries (e.g., 'chest pain in children', 'pediatric chest pain') rather than expanded conversational prompts (e.g., 'My child has chest pain, what should I do?'). This standardized input approach ensured that the generated responses reflected the models' raw, unguided linguistic complexity and content quality when handling direct medical search queries.

Limitations:
Additionally, search queries were submitted as direct keywords rather than conversational, scenario-based patient prompts. Because LLMs adapt their tone and readability based on prompt framing, future studies comparing direct search terms against expanded conversational questions could provide further insights into prompt-dependent readability variations.

Filtering of Search Terms: The criteria for removing the 8 terms that were eliminated when reducing the number of terms from 25 to 17 should be explained more clearly (with examples).

We thank the reviewer for this constructive suggestion.

To enhance methodological transparency, we have expanded the explanation regarding the exclusion criteria applied during the search term filtering process.

Out of the initial 25 search terms retrieved from Google Trends, 8 terms were systematically excluded based on three explicit criteria:
-Semantic Redundancy: Direct duplicates or lexical variations that yielded identical semantic content (e.g., "chest pain child" vs. "chest pain in children").
-Clinical Irrelevance / Off-Target Scope: Queries that pertained strictly to adult pathology, general pediatric wellness, or unrelated conditions without direct focus on pediatric chest pain symptoms (e.g., "chest pain in adults", "pediatric asthma").
High Vulnerability to Off-Context AI Outputs: Non-specific, broad phrases that led LLMs to generate non-clinical or ambiguous general content (e.g., "chest anatomy").

We have updated the text to clearly articulate these rules and provided concrete examples of the eliminated terms.

Revised Text
From an initial pool of 25 potential search terms, 8 were excluded based on predefined criteria, such as semantic redundancy, lack of alignment with the clinical boundaries of the study, or potential to generate off-context outputs in AI models [5,6,13]. Exactly 8 terms were excluded based on predefined methodological criteria: Semantic Redundancy (n=4): Lexical variations or morphological duplicates that yielded identical clinical topics were consolidated to prevent data duplication (e.g., 'chest pain child' and 'child chest pain' were excluded in favor of the primary high-volume term 'chest pain in children'). Out-of-Scope / Clinical Irrelevance (n=3): Queries targeting non-pediatric demographics or distinct non-cardiorespiratory pediatric symptoms were removed (e.g., 'adult chest pain causes', 'pediatric cough'). Off-Context Generative Risk (n=1): Broad anatomical terms that triggered generic educational descriptions rather than clinical/symptom-oriented evaluations were excluded (e.g., 'chest anatomy'). The remaining 17 distinct terms met all inclusion criteria by directly focusing on pediatric chest pain presentation, differential diagnoses, or related cardiac/non-cardiac etiology.

Two Different Calculators (Calculator 1 & 2): The text calculates and averages readability using two separate websites (readabilityformulas.com and online-utility.org). The algorithms used by these sites may show minor differences. A brief explanation should be given as to why a single standard tool or code library was not used instead of combining the results from the two sites.

We thank the reviewer for raising this thoughtful methodological point. Web-based readability engines often rely on slight variations in text preprocessing, word tokenization, and syllable-counting heuristics, which can introduce subtle computational discrepancies even when applying the same formula (e.g., FKGL or FRES).

Rather than relying on a single third-party web interface or script—which could inadvertently introduce platform-specific calculation bias or software-specific anomalies—we intentionally implemented a dual-engine cross-validation strategy. By independently running all outputs through two widely established digital platforms (*readabilityformulas.com* and *online-utility.org*) and utilizing their arithmetic averages, we aimed to:

1. Neutralize potential single-engine algorithmic variances and syllable-parsing errors.
2. Enhance internal validity and reproducibility through cross-platform verification.
3. Maintain consistency with previously validated medical informatics methodologies in readability literature.

We have updated the **Material and Methods** section under the *Textual Readability and Linguistic Complexity Assessment* subsection to explicitly clarify this rationale.

Revised Text:
To address potential algorithmic variations, syllable-tokenization discrepancies, and software-specific anomalies inherent in automated text parsers, a dual-platform cross-validation protocol was executed. All raw textual outputs were processed independently through two widely utilized digital engines: readabilityformulas.com (Calculator 1) and online-utility.org (Calculator 2). Using a single web tool or codebase risks introducing platform-specific measurement bias. Therefore, integrating data across two independent calculation interfaces and utilizing their arithmetic mean ensured cross-platform verification, minimized technical variance, and enhanced the overall methodological robustness and reproducibility of the readability indices.


3. Discussion and Conclusion: Clarity of the Main Message: Your finding of the "Readability-Quality Paradox" is the strongest aspect of the article. That is, Perplexity provides more accurate and scientific information, but its language is academic and difficult; ChatGPT/Gemini is easier to read, but its source/accuracy performance is lower. This message could be summarized more clearly and emphatically in the discussion section.

We express our sincere gratitude to the reviewer for recognizing the core insight of our work. The inverse relationship between linguistic accessibility and clinical accuracy—which we define as the **"Readability-Quality Paradox"**—is indeed the primary takeaway of this study.

As highlighted by the reviewer, while retrieval-augmented models like Perplexity excel in clinical rigor and source documentation, they produce outputs at a post-secondary reading level that exceeds general health literacy standards. Conversely, models like ChatGPT and Gemini generate more accessible text for the lay public, but at the expense of lower source reliability and quality metrics (DISCERN/JAMA scores).

To make this key finding more emphatic and central to our manuscript, we have revised the **Discussion** section by adding a dedicated subsection titled *"The Readability-Quality Paradox in AI-Generated Health Information."* We have also streamlined our concluding paragraphs to explicitly emphasize the clinical implications of this trade-off for patient education.

Revised TExt:
A key finding of this study is the 'Readability-Quality Paradox' observed in AI-generated health information. Our comparative analysis reveals a clear trade-off between textual accessibility and clinical reliability: while search-augmented models like Perplexity offer higher content quality and superior source attribution, they utilize complex academic language that exceeds standard health literacy thresholds. Conversely, models such as ChatGPT and Gemini produce simpler, more readable text for the general public, but achieve lower scores in clinical quality and reference verification. This paradox highlights a practical challenge for digital patient education, as the most accurate AI outputs remain difficult for average readers to comprehend, whereas the most accessible outputs carry lower clinical depth. Future medical AI applications must focus on bridging this gap by delivering high-fidelity clinical evidence within accessible language frameworks."

Clinical Recommendations: Practical advice for physicians and parents could be made more concrete with 1-2 sentences.

Thank you for this constructive suggestion. We have revised the tex to provide more concrete and concise practical advice for both physicians and parents in two focused sentences:

Revised Text:
Although our study showed that the texts provided by AI chatbots excelled in readability with ChatGPT-5 and in content quality and reliability with Perplexity due to scientific referencing, no matter how advanced artificial intelligence becomes, it can never replace face-to-face physician examination, one-on-one clinical evaluation, and physician-patient communication; these tools should only be considered for preliminary information purposes, and diagnosis and treatment should be based on direct guidance from expert physicians (x). Future bibliometric and altmetric in-depth analyses, methodological expansions, and comprehensive research integrated with clinical practice can provide a solid foundation for this area (y).
x. Doi: 10.1371/journal.pone.0353355.
y. Doi: 10.30621/jbachs.1096610


Decision: Minor Revision. The idea and dataset of the article are strong. The publication will be ready once the aforementioned language corrections, prompt clarifications are completed.

*We would like to thank all our reviewers for their valuable opinions and suggestions. We learned a lot from you..*
